# Navigation of frameless fixation for gamma knife radiosurgery using fixed augmented reality

**DOI:** 10.1038/s41598-022-08390-y

**Published:** 2022-03-16

**Authors:** Hyeong Cheol Moon, Sang Joon Park, Young Deok Kim, Kyung Min Kim, Ho Kang, Eun Jung Lee, Min-Sung Kim, Jin Wook Kim, Yong Hwy Kim, Chul-Kee Park, Young Gyu Kim, Yun-Sik Dho

**Affiliations:** 1 Department of Neurosurgery, Chungbuk National University Hospital, Cheongju, Republic of Korea; 2 Department of Neurosurgery, Chungbuk National University College of Medicine, Cheongju, Republic of Korea; 3 MEDICALIP Co. Ltd., Seoul, Republic of Korea; 4 Department of Neurosurgery, Seoul National University Hospital, Seoul National University College of Medicine, Seoul, Republic of Korea

**Keywords:** Translational research, Three-dimensional imaging

## Abstract

Augmented reality (AR) offers a new medical treatment approach. We aimed to evaluate frameless (mask) fixation navigation using a 3D-printed patient model with fixed-AR technology for gamma knife radiosurgery (GKRS). Fixed-AR navigation was developed using the inside-out method with visual inertial odometry algorithms, and the flexible Quick Response marker was created for object-feature recognition. Virtual 3D-patient models for AR-rendering were created via 3D-scanning utilizing TrueDepth and cone-beam computed tomography (CBCT) to generate a new GammaKnife Icon™ model. A 3D-printed patient model included fiducial markers, and virtual 3D-patient models were used to validate registration accuracy. Registration accuracy between initial frameless fixation and re-fixation navigated fixed-AR was validated through visualization and quantitative method. The quantitative method was validated through set-up errors, fiducial marker coordinates, and high-definition motion management (HDMM) values. A 3D-printed model and virtual models were correctly overlapped under frameless fixation. Virtual models from both 3D-scanning and CBCT were enough to tolerate the navigated frameless re-fixation. Although the CBCT virtual model consistently delivered more accurate results, 3D-scanning was sufficient. Frameless re-fixation accuracy navigated in virtual models had mean set-up errors within 1 mm and 1.5° in all axes. Mean fiducial marker differences from coordinates in virtual models were within 2.5 mm in all axes, and mean 3D errors were within 3 mm. Mean HDMM difference values in virtual models were within 1.5 mm of initial HDMM values. The variability from navigation fixed-AR is enough to consider repositioning frameless fixation without CBCT scanning for treating patients fractionated with large multiple metastases lesions (> 3 cm) who have difficulty enduring long beam-on time. This system could be applied to novel GKRS navigation for frameless fixation with reduced preparation time.

## Introduction

Augmented reality (AR) is an advanced technology that mixes the virtual world with the real world in different proportions^1^. It has found good potential applications in many fields, such as military training, entertainment, manufacturing, and medical in recent years. In the neurosurgery field, AR is used as a volumetric image guide^2,3^ and phone-based neurosurgical navigation system^4–6^. AR navigation uses various devices such as smartphones, desktop PCs, head-mounted displays, and AR glasses. The AR system commonly consists of an outside-in method using sensors either attached to the computer, head-mounted display, or pre-installed, which can be operated intuitively in conjunction with hand movements^7^, but sensor recognition could go out-of-range due to misalignment after initial registration. The inside-out tracking method can detect a continuous tracing of the target from the user’s location through cameras and sensors mounted simultaneously on the device used for visualization^8^. Although inside-out tracking has less accuracy compared to outside-in tracking, installing the equipment for visualization and object detection for registration is low cost. We previously reported on an inside-out tracking-based AR-neuro-navigation system using ARKit® (Apple Inc.) based software^9^. We applied the inside-out tracking for radiosurgery by mounting it to a 4th generation iPad Pro (Apple Inc. San Francisco, USA) to test the feasibility to develop clinically usable inside-out tracking AR in gamma knife radiosurgery (GKRS), which utilizes devices in a fixed-stated, called fixed-AR.

The high dose of radiation delivered through GKRS requires a high degree of accuracy and immobilization of the head^10,11^. Accuracy with head-frame fixation is essential to limit irradiation of the surrounding anatomical structures^12^. Head-frame placement is invasive involving screw fixation at four specific points in the patient’s skull and difficult for fractionated treatment of large lesions^11,13^. The latest version in GammaKnife (GK) Icon™ is capable of non-invasive fixation and fractionated treatment by utilizing cone-beam computed tomography (CBCT) and detecting the motion by high-definition motion monitoring (HDMM). CBCT could be acquired by either a higher signal (CTDI 6.3) preset or lower dose (CTDI 2.5) and registered with the stereotactically-defined image set for comparison between patient coordinates at the time of treatment imaging; the HDMM system can currently be used for head immobilization with a thermoplastic mask instead of a head-frame^14^. It is the frameless fixation of the GK Icon™. During subsequent delivery of the adapted treatment plan from the HDMM system, it tracks the displacement of the patient’s nose marker related to the four immobile reflectors fixed to the Icon™ head support system in real time^15^. However, the irradiation is executed only when the magnitude of displacement returns under the threshold; if the threshold is exceeded, the new CBCT scan is processed to allow coordinates acquire a new position. Wright et al*.* suggested that the target and nose marker typically varies throughout a clinically relevant extent of stereotactic space, and the average HDMM threshold of 1.4 mm may be appreciated for 41 volumes^15^. However, patients with multiple metastases or older age seem to be intolerant to the lower threshold according to the increased beam-on time. Kim et al. reported that the elapsed beam-on time, including beam-paused time due to motion of the patient, defines the tolerance for around 30 min (min) in older patients (> 65 years)^16^. Thus, keeping the appropriate HDMM threshold without the new position for CBCT scanning is important for intolerant patients in GKRS frameless fixation. The navigation of patient positioning under frameless fixation is useful to reduce the preparation time and unnecessary CBCT scanning. If we used CBCT images to make a 3-dimensional (3D)-virtual model using fixed-AR, the frameless fixation could possibly be repositioned, guided by a 3D-virtual model, based on initial planning for CBCT without unnecessary CBCT scanning.

3D-scanning increases the accuracy and makes it easy to obtain the virtual 3D-model. Recently, TrueDepth technology in the latest devices by Apple Inc. is used for measuring the task with accuracies in the millimeter range. TrueDepth uses vertical-cavity surface-emitting laser (VCSEL) technology and consists of an infrared camera, a conventional camera, proximity sensor, spot projector, and flood illuminator^17^. The front-facing camera provides the depth data in real-time along with visual information, and the system uses a light-emitting diode to project an irregular grid of over 30,000 infrared dots to record the depth within milliseconds. To scan objects, an additional application was installed^18^. Although Heges application was evaluated with the finest 3D resolutions under 0.5 mm^17^, the accuracy is affected by the scanning strategy and post-processing^19^. The potential of TrueDepth in the recent iPad Pro as 3D-scanning in Heges application was evaluated using fixed-AR in GKRS.

To apply this new AR technology for GKRS, the virtual models were established using existing planning CBCT images, and a novel TrueDepth 3D-scanning method. In this study, we investigated the navigation of frameless fixation using fixed-AR with the virtual models of CBCT scans and 3D-scans into a 3D-printed patient model for GKRS.

## Results

### Execution of fixed-AR navigation in frameless fixation

**Figure 1:**
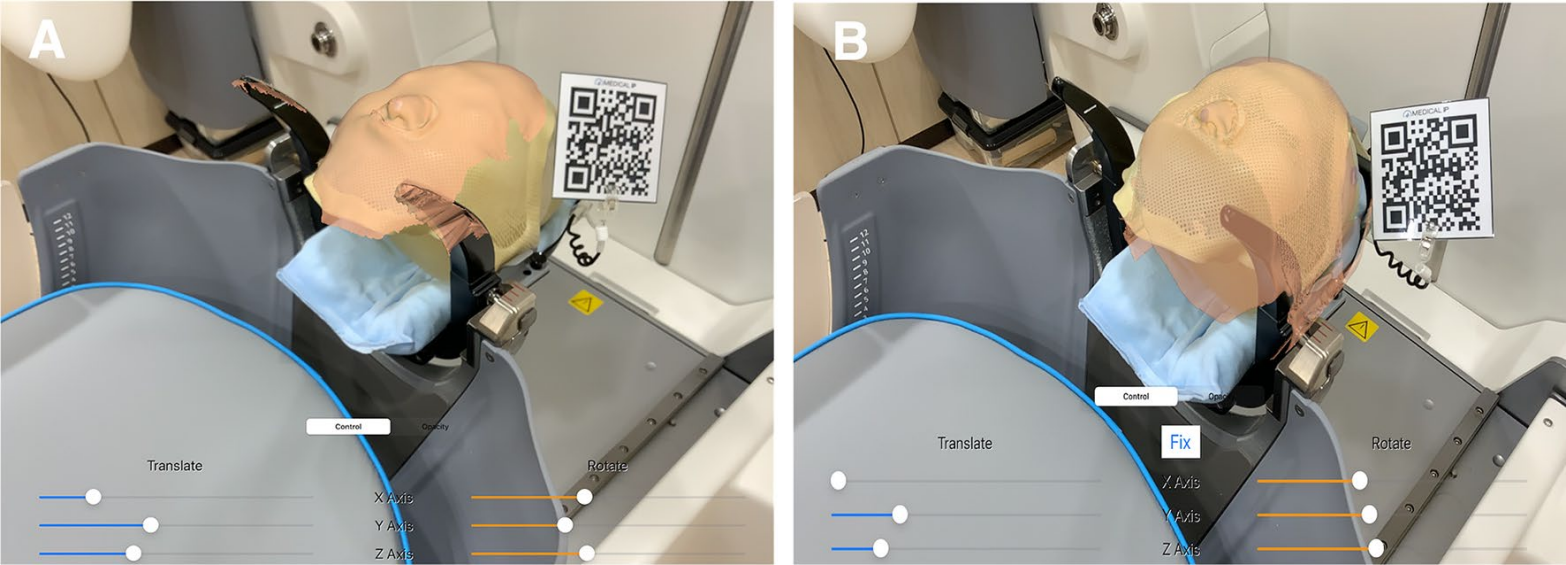
The virtual models and a 3D-printed model overlap correctly using fixed-augmented reality. The virtual model based on 3D-scanning (**A**) and that based on cone-beam computed tomography (**B**) are shown.

When the navigation of frameless fixation was executed through the new application, it overlapped on the 3D-printed model. To complement the fixed device state, the quick response (QR) marker attached to the mask indicator and iPad Pro was installed to the cradle beside a couch bed. The QR marker was designed for various directions by adjusting the registration target. Fixed-AR navigation is confirmed to correctly overlap with the 3D-printed patient model and virtual models based on 3D-scanning and CBCT in Fig. 1.

### Validation of frameless fixation based on virtual models using Fixed-AR

Rotational and translational set-up errors for frameless fixation based on virtual models using fixed-AR are summarized in Table 1. The mean rotational errors for the virtual models were small in all axes, less than 1.0° except for the Y-axis (1.36 ± 1.064°) in the 3D-scanning virtual model. The mean translation error for the virtual models was less than 1 mm in all axes.

**Table 1:** The mean translational and rotational set-up errors in frameless fixation using fixed-AR.

	Planning CBCT + pretreatment CBCT	Planning CBCT + Virtual model of 3D-scanning with fixed-AR	Planning CBCT + Virtual model of CBCT with fixed-AR
**Rotation (°)**			
x-axis	0.010 ± 0.010	0.313 ± 0.364	0.387 ± 0.523
y-axis	0.007 ± 0.012	1.360 ± 1.064	0.880 ± 1.060
z-axis	0.013 ± 0.06	0.743 ± 0.801	0.587 ± 0.611
**Translational (mm)**			
x-axis	0.027 ± 0.029	0.403 ± 0.038	0.677 ± 0.422
y-axis	0.040 ± 0.010	0.190 ± 0.135	0.297 ± 0.249
z-axis	0.013 ± 0.015	0.633 ± 0.215	0.820 ± 0.887

The setup errors for frameless fixation are based on virtual models followed by co-registration with planning CBCT. All data are shown as mean ± standard deviation.

The fiducial markers in the 3D-printed model are defined in seven points out of eight points because of the exceeding image definition from CBCT scanning. The mean error of fiducial marker coordinates for frameless fixation based on virtual models in fixed-AR are summarized in Table 2. Comparison of the planning CBCT with pretreatment CBCT revealed all mean errors were under 1.0 mm. However, the planning CBCT with virtual models had a mean error of 0.28 to 2.72 mm, including 3D errors.

**Table 2:** The mean errors of fiducial marker coordinates in virtual models with fixed-AR.

Location of fiducial markers	Planning CBCT + pretreatment CBCT				Planning CBCT + Virtual model of 3D-scanning with fixed-AR				Planning CBCT + Virtual model of CBCT with fixed-AR			
	Δx (mm)	Δy (mm)	Δz (mm)	Δr (mm)	Δx (mm)	Δy (mm)	Δz (mm)	Δr (mm)	Δx (mm)	Δy (mm)	Δz (mm)	Δr (mm)
Left frontal	0.48 ± 0.11	0.52 ± 0.17	0.35 ± 0.11	0.90 ± 0.20	1.31 ± 0.85	0.99 ± 0.51	2.15 ± 1.42	2.71 ± 1.75	1.16 ± 1.20	0.71 ± 0.30	1.33 ± 1.48	2.10 ± 1.59
Right frontal	0.29 ± 0.19	0.44 ± 0.19	0.14 ± 0.07	0.75 ± 0.30	1.35 ± 1.11	1.29 ± 0.97	1.77 ± 0.83	2.72 ± 1.34	1.29 ± 1.38	0.95 ± 0.62	1.01 ± 0.98	2.22 ± 1.14
Left parietal	0.44 ± 0.43	0.24 ± 0.14	0.14 ± 0.13	0.68 ± 0.21	0.57 ± 0.50	0.76 ± 0.61	2.32 ± 0.68	2.63 ± 0.43	0.90 ± 1.03	0.94 ± 0.37	2.04 ± 0.40	2.57 ± 0.47
Right parietal	0.29 ± 0.11	0.50 ± 0.41	0.05 ± 0.03	0.59 ± 0.32	0.49 ± 0.29	1.38 ± 0.57	1.73 ± 1.41	2.38 ± 1.26	1.50 ± 0.75	0.82 ± 0.77	1.73 ± 1.18	2.58 ± 1.24
Superior parietal	0.40 ± 0.36	0.50 ± 0.26	0.34 ± 0.27	0.73 ± 0.22	1.85 ± 1.61	0.79 ± 0.73	0.99 ± 0.90	2.72 ± 0.63	1.25 ± 0.77	1.56 ± 0.91	0.84 ± 0.73	2.39 ± 0.71
Inferior parietal	0.45 ± 0.40	0.39 ± 0.05	0.42 ± 0.27	0.78 ± 0.09	1.15 ± 0.14	0.55 ± 0.74	0.78 ± 0.68	1.65 ± 0.53	1.74 ± 1.16	0.85 ± 0.69	1.37 ± 0.57	2.44 ± 1.30
Posterior occipital	0.39 ± 0.33	0.39 ± 0.34	0.26 ± 0.20	0.71 ± 0.19	1.53 ± 0.99	0.54 ± 0.13	0.75 ± 0.54	1.93 ± 0.70	1.23 ± 0.96	0.28 ± 0.29	1.16 ± 0.89	1.92 ± 0.91

The mean error of fiducial markers is calculated by CBCT scanning coordinates (X,Y,Z). All data are shown as mean ± standard deviation.

For patients undergoing GKRS treatment with frameless fixation for a few days, we measured the HDMM differences between initial fixation and re-fixation, navigated by the virtual models for three days. The mean differences in the virtual model of 3D-scanning and CBCT were 1.19 ± 0.32 mm and 1.21 ± 0.02 mm, respectively. The re-fixation navigated by the virtual models without CBCT revealed HDMM values under 1.5 mm.

The AR-guided initial preparation time was approximately 8 min whereas the CBCT rescan was approximately 5 min. If we initially set the fixed-AR, it would take only 1 min to repeat the GKRS procedure.

### Optimization of frameless fixation with correction function

In cases of inaccurate registration after fixed-AR navigation, the user could use the correction function to adjust the surface position of the fixed-AR navigation that accurately overlaps with the nose marker in GKRS. Both 3D rendering in 3D scanning and CBCT distorted from registration were adjusted to the correct position using the correction function.

## Discussion

GKRS requires an accurate and precise high-dose radiation for a specific target while minimizing the potential radiation toxicity for the surrounding tissue. Recently, the GK Icon™ version is capable of frameless (mask) fixation using CBCT, which is used to verify the patient position during set-up prior to irradiation^20^. Frameless fixation is widely used for large lesions with hypofractionated treatment; it is important to endure the tolerance and long beam-on-time, allowing immobilization of the patient’s head. If AR navigation using virtual models replace the patient’s head position navigation, maintaining the appropriate HDMM threshold without repositioning CBCT scanning will be accomplished more easily with the reducing of the preparation time and the irradiation due to CBCT scanning in GKRS. This study represented the mean set-up errors within 1.5°, and 1 mm in both methods. The mean differences of fiducial markers from coordinates were within 2.5 mm, and the 3D errors were within 3 mm in both methods. Our fiducial marker error results were larger than set-up errors; however, the mean errors determined by manual measurement are generally acceptable within the 3 mm threshold. The mean differences of HDMM values were within 1.5 mm compared to initial HDMM values.

We demonstrated that virtual models with fixed-AR could be applied for intolerant patients with frameless fixation, requiring repeated CBCT scanning to exceed the HDMM threshold.

Patient-specific planning based on individual characteristics and conditions is important for precise treatment in neurosurgery^21,22^. In radiosurgery, all patients have various lesions including malignant or benign tumors, vascular disease, and functional diseases. In our country, demographic projections for older adults have increased, implying a consequent increase in cancer incidence and mortality in this population^23^. Radiosurgery delivered by highly focusing radiation with sharp dose fall-off is theoretically expected to reduce delayed neurotoxicity^24^. Recently, hypofractionated radiosurgery was found to be effective as a single-session radiosurgery with minimal toxicity for large brain metastases (> 10 cm^3^)^25,26^. However, the patients with multiple metastases or older age seem to be intolerant to the lower HDMM values according to increased beam-on time. Thus, keeping the appropriate HDMM threshold without a new CBCT scanning position is important for intolerant patients in frameless fixation in GKRS. In case of radiation exposure, although CBCT (2.5 mGy or 6.4 mGy) is a low dose CT, CBCT scans still involve radiation exposure. Furthermore, repeated CBCT scanning could require continual CBCT data storage. CBCT data is approximately 200 MB, the virtual model 3D data is approximately 80 MB. The virtual model 3D data requires less storage capacity and replace CBCT rescan data.

The scanning accuracy decides the potential use of 3D scanner applications. The iOS of Apple’s smartphones and tablets provides 3D data without the operator’s measurement experiences, called TrueDepth Scanner, based on the structure-light principle^27^. TrueDepth-based 3D scanning reveals the highest deviations in cylindricity (0.82 mm on average) and roundness (1.17 mm on average)^17^. In another 3D scanning study, Camison et al. demonstrated that the points calculated from a total of 136 distances had an average deviation (mm) of 0.84^28^. Our results revealed that translational errors were less than 1.00 mm in all axes in virtual models. The rotational error in the Y-axis was only higher in the virtual model of 3D-scanning compared to that of CBCT. However, all fiducial marker errors were under 3 mm without CBCT scanning. These results could be applied to the patients with metastases, long beam-on-time, and no eloquent areas. Paul et al. demonstrated that when using 3 mm as a cut-off there was no effect on local recurrences identified ^29^.

The novel method of inside-out fixed AR is performed by a physician or physicist experienced in GKRS to match the nose marker target under frameless fixation based on user-determined registration with the 3D-printed patient model and virtual models. The fixed-AR navigation do not execute the automatic registration into frameless fixation; however, the position of fixed-AR images could be adjusted using the correction function into a fixated-state in a frameless adaptor. We recommend that the navigation of the fixed-AR system could be utilized simultaneously along with the frameless-fixation before pretreatment CBCT scanning. If a patient cannot endure the long beam-on time, frameless re-fixation without CBCT can be performed with a short resting period using the fixed-AR system. Frameless fixation can be maintained for a long time, which may result in pressure on the face and make the patient feel uncomfortable. For this reason, it is important to conduct the frameless fixation close to patient's initial position. The AR navigation has the potential capability for real-time monitoring of a patient’s movement in GKRS. The detection of the patient’s movement only depends on the motion marker in GK Icon™; however, if the real-time monitoring is possible in AR navigation, frameless GKRS can be performed without a mask or with the mask loosely fixed in the future.

This study has a few limitations. The fixed-AR system is not automatically registered to the object; it takes a few attempts to overlap the object and virtual models. The development of a fixed-AR system should be considered to register AR automatically into the object using real-time tracking, and we are planning to build a storage server with patient specific information, including virtual models in the fixed-AR application in order to conduct a clinical trial in the future. Although the virtual model of 3D-scanning consists of a fixed-AR system, it is still inaccurate to register it to the entire real object. 3D-scanning accuracy will determine the potential applications of 3D-scanners. To guide  the surface fixed-state with 3D-scanning, the general 3D-scanning accuracy of the entire object should be evaluated.

## Conclusions

We demonstrated that fixed-AR navigation is a useful tool for frameless fixation without CBCT for GKRS. This method using conventional equipment and fixed-AR with inside-out tracking could be directly adapted to GKRS. Overall, when planning for small lesions or eloquent areas, frameless fixation and repositioning with CBCT scanning should be considered. However, in cases of patients with large metastases, no eloquent area and continual movements under frameless fixation could be navigated well with re-fixation and repositioning using a fixed-AR system without CBCT scanning.

## Methods

### 3D-printed patient model and initial frameless fixation

**Figure 2:**
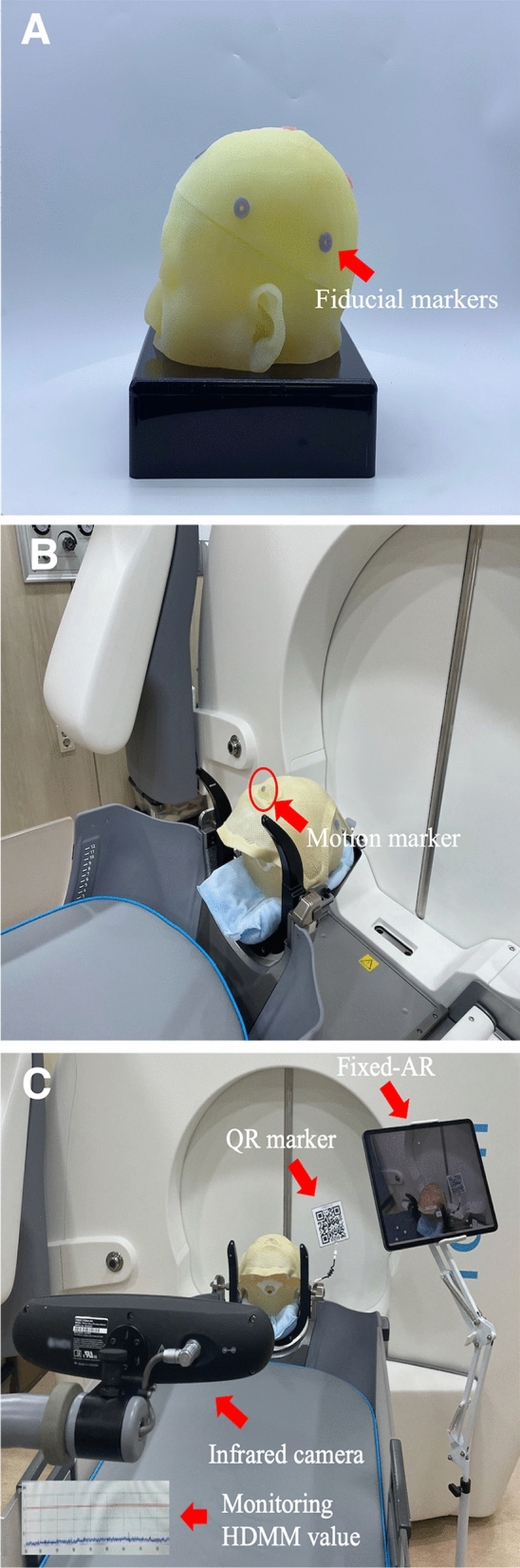
The fixed-AR execution prepared in the frameless fixation adaptor for GKRS. The 3D-printed patient model included fiducial markers (**A**). The 3D-printed model had a frameless adaptor with the motion marker (**B**). Implemented fixed-AR with the QR marker being monitored under the infrared camera (**C**). Quick Response, QR; Augmented Reality, AR; high-definition motion monitoring, HDMM.

The 3D-printed patient model was produced in the following three stages: 1) creation of a stereolithography (STL) file for 3D printing; 2) printing physibles using a 3D printer; and 3) post-processing performed via manual editing. The process was approved by the institutional review board (Seoul National University Hospital, IRB No. 1811–040-986 and Chungbuk National University Hospital, IRB No. 2019–06-015-001) and used to validate set-up errors, fiducial marker coordinates, and HDMM values. The IRBs of two institutions where a case originated waived the requirement for informed consent for using simulated patient’s phantom since there was no interaction with the patient. The 3D-printed patient model had a frameless adapter with a head cushion and was fixed with a thermoplastic Nanor® mask (Elekta instrument AB, Stockholm, Sweden). In the fixed-AR setting, we measured the initial fiducial marker coordinates after planning CBCT and HDMM values, which kept the motion value for 10 min, as presented in Fig. 2.

### 3D scanning and planning CBCT

The 3D-printed patient model with frameless fixation was taken for TrueDepth scanning using Heges application with iPad Pro. The STL scan file of the scan was imported to Meshmixer version 3.5 (https://www.meshmixer.com) to trim the background scanning, and smoothing processes were performed to improve the pixilation and obtain more uniform triangles.

**Figure 3:**
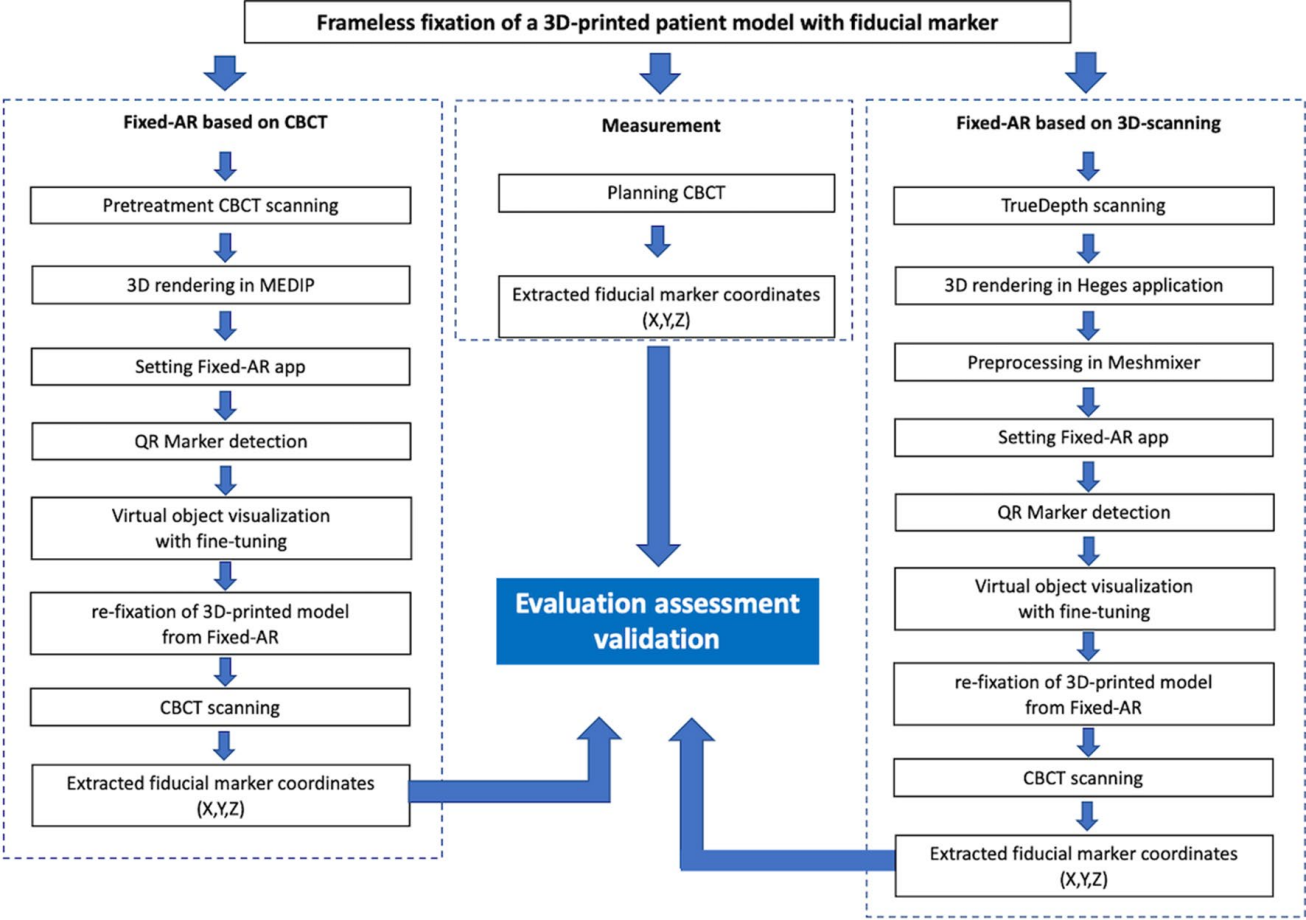
Schematic illustration of the procedure to evaluate frameless fixation for GKRS.

The 3D-printed patient model with frameless fixation was also scanned for planning CBCT scanning using Leksell GammaPlan Version 11.1 (Elekta instrument AB, Stockholm, Sweden), and exported CBCT Digital Imaging and Communications in Medicine (DICOM) files. The authors developed and released the software (MEDIP, https://medicalip.com/Download, MEDICALIP). The DICOM files were imported to MEDIP software for 3D-rendering, trimming the background, and smoothing processing. The MEDIP software is easy to operate due to its machine learning-based semi-automated segmentation function^30^. Thus, the 3D data can be prepared by medical staff without computer expertise. This procedure is presented in Fig. 3.

### Inside-out AR navigation and running fixed-AR

**Figure 4:**
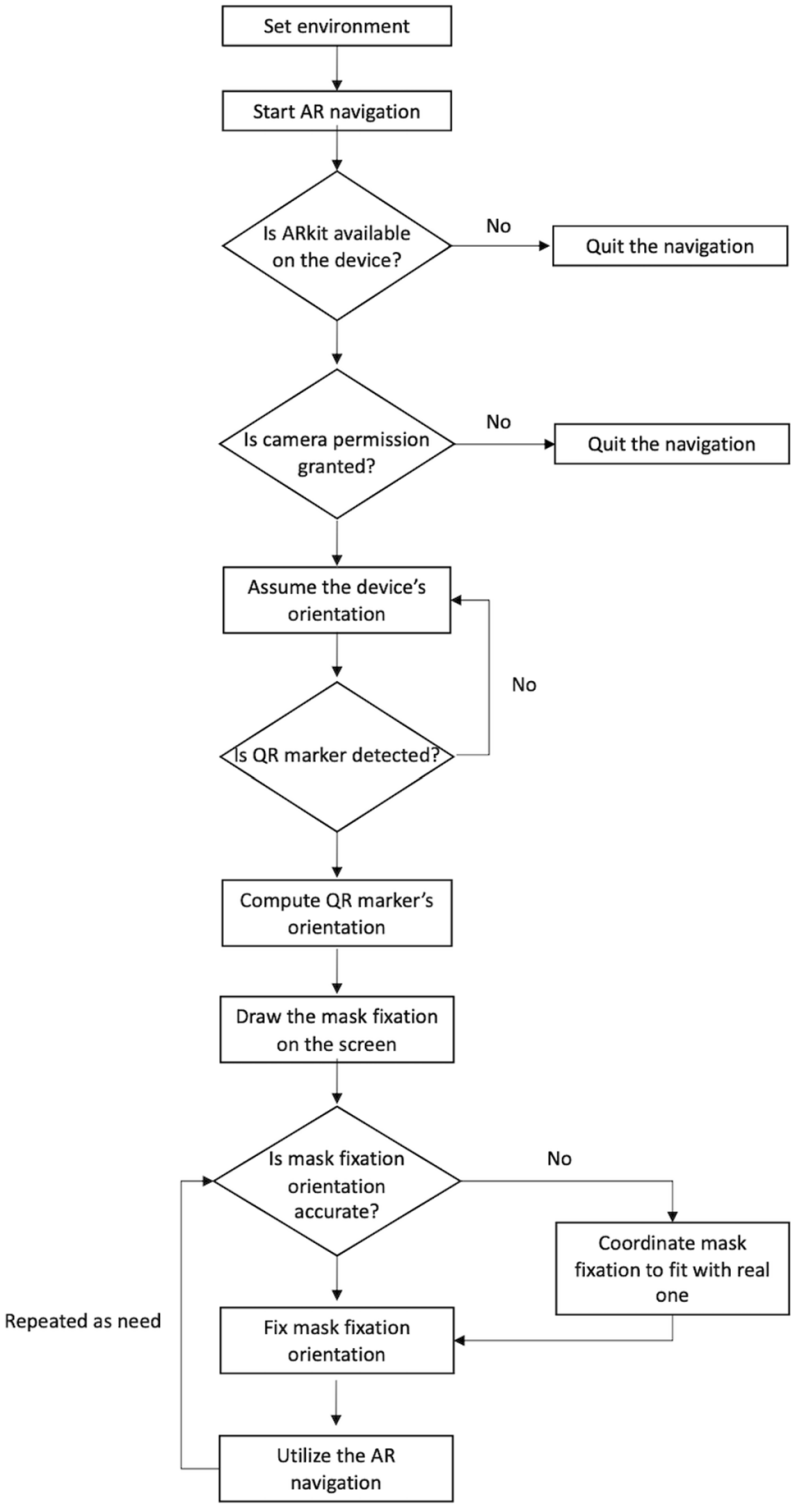
Workflow of the inside-out AR navigation algorithm and running fixed-AR.

The inside-out AR navigation (METAMEDIP navigation) was developed using ARkit® (Apple Inc.). It has not been released yet, but the technical methods were described in the previous study^30^. The inside-out AR navigation was processed in three steps: 1) device recognition is visualized, followed by 2) QR marker recognition, and 3) AR implementation and registration within the running environment. The workflow of the inside-out AR navigation algorithm and running fixed-AR is presented in Fig. 4. The QR marker is attached to the right mask fixation button adjacent to the matching target that has minimal light reflection and is unlikely to be easily obscured by other objects. The ARkit® is based on Visual Inertial Odometry (VIO), which measures the device location from inertial measurement unit (IMU)-based data that has a fast collection and calibrates using camera images. Moreover, ARkit® supports close-loop processing that corrects the trajectory by matching the trajectory with the starting point when moving the device and returning to the starting point during calculation with the VIO algorithms. This is collectively referred to as visual inertial simultaneous localization and mapping. The loop-closure process can be omitted due to the nature of this AR system wherein the device operates in a fixed state.

The QR marker images are used for estimating the position recognized by the camera in the feature detection algorithm, which commonly considers the corner and intersection of lines or the part with clear color contrasts (Black and White) as a feature point. After the device position is determined, the QR marker, recognized by the scale of objects in virtual space, is determined by calculating the distance between the device and the QR marker from the size of the recognized marker in the preceding step. After all the requisite data are calculated, the 3D scanning or CBCT-based virtual models are displayed at the designated positions from the marker for confirmation by the user. In case of errors in the automatically processed pre-registration, the user can correct the registration error by adjusting the position of the 3D virtual model using the correction function and then fix the position of the models to complete the registration.

### Validating the fixed-AR navigation registration and preparation time

The registration accuracy was measured using the following three methods: intuitive validation through visualization; set-up errors; and quantitative validation. The 3D-printed patient model was created and matched with 3D virtual models. Set-up errors were assessed by comparing the planning and pretreatment CBCTs^11^, which navigated to re-fixation using the fixed-AR system with the virtual models. Set-up error was defined as displacement of the skull in the stereotactic space. Setup errors were investigated by translational (mm) and rotation (°) methods for three days. The registration accuracy of the fiducial marker was validated by coordinating pretreatment CBCT, scanned from navigated re-fixation by the fixed-AR, to planning CBCT. The fiducial markers were attached to both hemispheres of the 3D-printed patient model in 8 points.

We defined the error of coordinates as follows:^31^.

Δx = x-axis coordinate error in planning CBCT + pretreatment CBCT or virtual models.

Δy = y-axis coordinate error in planning CBCT + pretreatment CBCT or virtual models.

Δz = z-axis coordinate error in planning CBCT + pretreatment CBCT or virtual models.

Furthermore, the 3D error (Δr) was defined as a localization error by the following formula:

$$\Delta {\text{r}} = \surd \left( {\Delta {\text{x}}^{{2}} + \Delta {\text{y}}^{{2}} + \Delta {\text{z}}^{{2}} } \right).$$

The differences of HDMM values were investigated  in initial fixation and re-fixation navigated fixed-AR. The mean accuracy was evaluated by measuring the X, Y, and Z coordinates for three days. We also measured the preparation time from setup to GKRS procedure between the AR guided re-fixation and the CBCT rescan.

## Correction function of misalignment with fixed-AR

For cases when automatic registration using the QR marker could be misaligned, the correction function of fixed-AR was developed and could alter the location of the virtual 3D model of AR navigation based on the following principles^9^: the virtual space is shown on the device screen through the coordinates (local, world, and camera); the local coordinate is a part of the virtual 3D model itself. The world coordinate refers to the virtual space wherein the model is placed, while the camera coordinate refers to the coordinate system wherein the world coordinate is viewed from the standard reference of the camera. The correction function is based on the world coordinate, and the movement is based on the camera coordinate, which assists the user in determining the desired direction and the rotation of movement intuitively.

Also, the opacity of the virtual models can be adjusted by using opacity-adjustment function. These functions allow AR implementation by further emphasizing the objects.

## Data Availability

The datasets used in this study are available from the corresponding author on reasonable request.
